# Genetic polymorphisms of Bcl-2 promoter in cancer susceptibility and prognosis: a meta-analysis

**DOI:** 10.18632/oncotarget.15751

**Published:** 2017-02-27

**Authors:** Zhongqiang Yao, Binhui Yang, Zhongqiu Liu, Wei Li, Qihua He, Xingchun Peng

**Affiliations:** ^1^ Department of Medical Oncology, 3201 Affiliated Hospital of Medical College of Xi’an Jiaotong University, Hanzhong, 723000, Shanxi Province, P. R. China; ^2^ Department of Centre of Oncology, Renmin Hospital, Hubei University of Medicine, Shiyan, 442000, Hubei Province, P. R. China

**Keywords:** Bcl-2, cancer, meta-analysis, case-control studies

## Abstract

Bcl-2 is critical for tumorigenesis. However, previous studies on the association of Bcl-2 promoter polymorphisms with predisposition to different cancer types are somewhat contradictory. Therefore, we performed this meta-analysis regarding the relationship between Bcl-2 promoter single nucleotide polymorphisms (SNPs) and cancer susceptibility and prognosis. Up to August 2016, 32 original publications were identified covering two Bcl-2 promoter SNPs (rs2279115 and rs1801018). Our results showed statistically significant association between rs2279115 and cancer susceptibility and prognosis in all four genetic models but not in rs1801018. Subgroups analysis indicated that rs2279115 was associated with a significantly higher risk of cancer susceptibility in Asia but not in Caucasian. Furthermore, rs2279115 was associated with a significantly higher risk in digestive system cancer and endocrine system cancer but not in breast cancer, respiratory cancer and hematopoietic cancer. Simultaneously, rs2279115 was correlated with a significantly higher risk of cancer prognosis in Asia but not in Caucasian. Considering these promising results, rs2279115 may be a tumor marker for cancertherapy in Asia. Sensitivity analysis show four gene model were stable, and no publication bias was observed in all four gene model. Large sample size, different ethnic population and different cancer type are warranted to validate this association.

## INTRODUCTION

Apoptosis plays an important role in cell fate and homeostasis which is a critically in biological process [1]. B cell lymphoma-2 (Bcl-2) is a important protein in apoptotic pathway, and is one of the most important oncogenes in the study of apoptosis. Bcl-2 mainly located on the mitochondrial outer membrane, or by the signal stimulation after the transfer to the mitochondrial outer membrane [2]. Currently research shows that cancer, neurodegenerative disorders, ischemia and autoimmune diseases are associated with Bcl-2 function abnormalities [3–5]. High expression of Bcl-2 is associated with different cancer types, and has been reported in esophageal cancer, non-small cell lung cancer, endometrial cancer, breast cancer, prostate cancer, lung cancer, chronic lymphocytic leukemia, diffuse large B-cell lymphoma etc [6–10]. There is increasing evidence that Bcl-2 gene polymorphism may be associated with cancer susceptibility and prognosis.

Human Bcl-2, located on chromosome 18q21.3, consists of two promoters, which called promoter 1 (P1) and promoter 2 (P2) [11]. P1 and P2 have different functions. More than 95% of the BCL-2 transcription is started by P1, it is at the initial point of about 1.7 KB upstream of translation, which has no typical TATA box, but have rich in GC box that can be combined with Sp-1. Also, P1 driven transcription mainly from GC box near the beginning, this is very similar with other housekeeping gene promoter. The chromosome structure of P1 analysis showed that it could be a constitutive promoter. P2 located downstream of the P1 at about 1.3kb (at translation starting point about 80bp upstream). Compared with P1, P2 is mainly an inducible promoter, and a small part of Bcl-2 transcription is driven by the P2 [12, 13]. Previous studies have identified rs2279115 which located in P2 promoter [14] have conflicting results between cancer susceptibility [15–36] and prognosis [15, 18, 19, 30, 37–46]. Furthermore, the correlation between rs1801018 single nucleotide polymorphisms (SNPs) and cancer susceptibility are somewhat contradictory [29, 30, 33, 36, 47].

To confirm whether Bcl-2 promoter polymorphisms are related to cancer, we performed this meta-analysis, aiming to measure the correlation between Bcl-2 promoter polymorphisms and cancer susceptibility and prognosis.

## RESULTS

### Studies retrieved and characteristics

**Figure 1:**
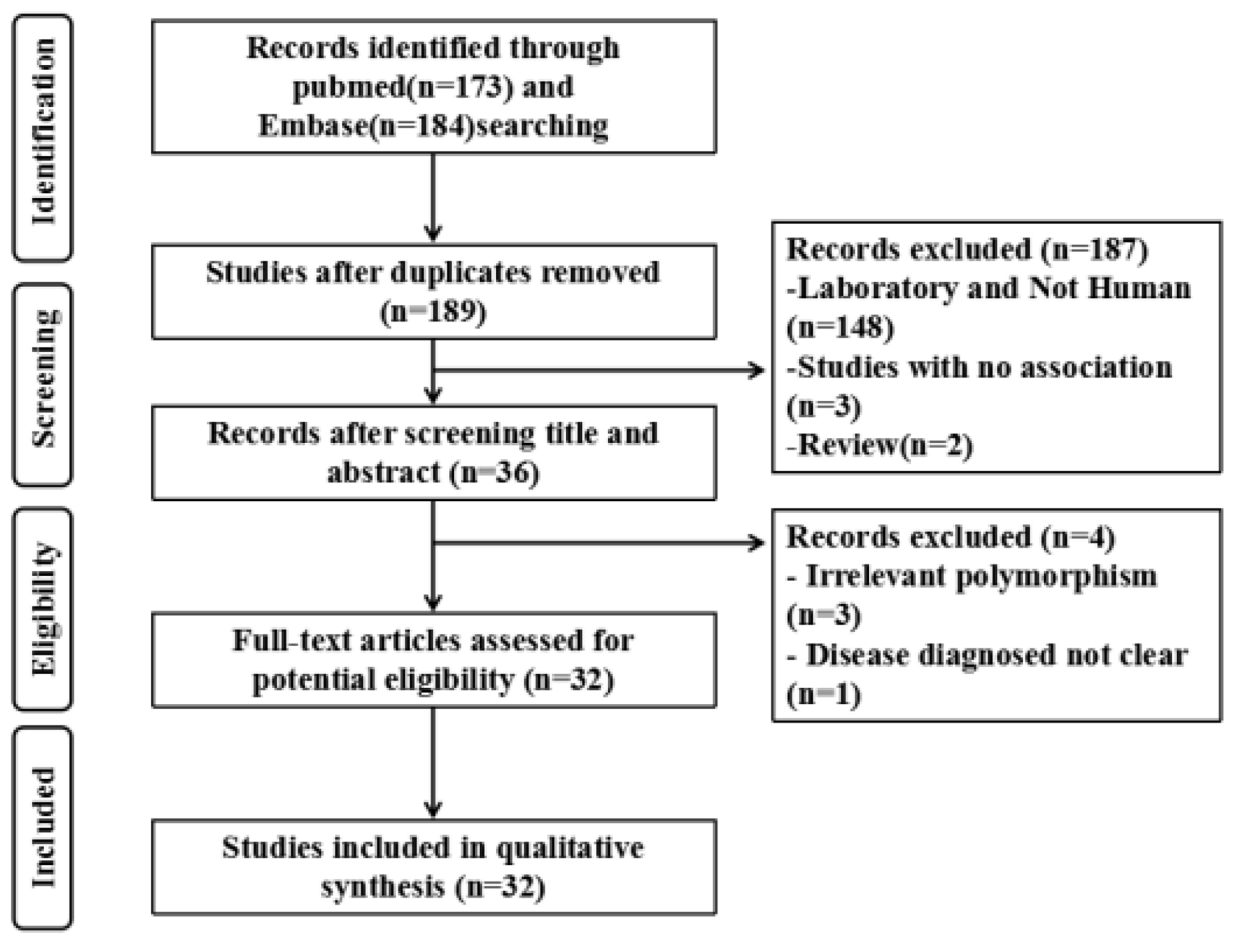
Flow diagram of the study selection process

Following an initial search, after duplicates removed, 189 studies were retrieved (PubMed: 173, Embase: 184). 6 review and 151 irrelevant studies and were excluded. Finally, 32 studies (6950 cases and 7984 controls) were chosen, and the data were extracted. A flow chart were carefully identified of the search process in Figure 1. The departure of Bcl-2 promoter polymorphisms frequencies from expectation under Hardy-Weinberg equilibrium (HWE) was assessed by chi-square in control group, and it was considered to be disequilibrium if P< 0.05. Six studies were excluded by HWE expectation (P< 0.05). The genotype distributions of all studies are summarized in Supplementary Table 1-3.

Overall, eighteen studies evaluating rs2279115 (6950 cases and 7984 controls) polymorphism in cancer susceptibility, three studies (1260 and 1440) evaluating rs1801018 polymorphism in cancer susceptibility, thirteen studies (4013 and 5319) evaluating rs2279115 polymorphism in cancer prognosis, and five studies (1889 and 2110) evaluating rs1801018 polymorphism in cancer prognosis. As for studies investigating the association between rs2279115 polymorphism and cancer susceptibility in cancer type, two studies evaluating rs2279115 polymorphism in cancer susceptibility in hematopoietic cancer and endocrine system cancer, three studies evaluating rs2279115 polymorphism in cancer susceptibility in digestive system cancers and respiratory cancer, four studies evaluating rs2279115 polymorphism in cancer susceptibility in breast Cancer. As for studies investigating the association between rs2279115 polymorphism and cancer susceptibility in ethnicity, eleven studies evaluating rs2279115 polymorphism in cancer susceptibility in Asia, seven studies evaluating rs2279115 polymorphism in cancer susceptibility in Caucasian. As for studies investigating the association between rs2279115 polymorphism and cancer prognostic in ethnicity, six studies evaluating rs2279115 polymorphism in cancer prognostic in Asia, seven studies evaluating rs2279115 polymorphism in cancer prognostic in Caucasian. Furthermore, three studies evaluating rs1801018 polymorphism in cancer susceptibility in Asia, and five studies evaluating rs1801018 polymorphism in cancer prognostic.

### Meta-analysis of BCL-2 promoter polymorphisms and cancer susceptibility

Overall, our results showed that rs2279115 was correlated with a significantly higher risk of cancer susceptibility in allelic, dominant, recessive, and additive models(OR= 1.16, 95% CI:1.05,1.29, P= 0.004, allelic models respectively). However, rs1801018 had no correlation with the risk of cancer susceptibility in allelic, dominant, recessive, and additive models(OR= 1.48, 95% CI:0.90,2.44, P= 0.119, allelic model respectively) in Table 1.

**Table 1:** Meta-analysis of BCL-2 promoter polymorphisms and cancer risk

	No.ofstudies	*P*_Q_	I^2^	OR	95% CI	*P*_Z_	Model
**rs2279115**	**18(6950/7984)**						
C vs. A		0.000	74.4%	1.16	1.05,1.29	0.004	Random-effects model
CC+ CA vs. AA		0.000	65.3%	1.19	1.01,1.41	0.039	Random-effects model
CC vs. CA+AA		0.000	67.5%	1.23	1.08,1.41	0.002	Random-effects model
CC vs. CA		0.004	53.8%	1.20	1.07,1.35	0.003	Random-effects model
**rs1801018**	**3(1260/1440)**						
A vs. G		0.034	70.4%	1.48	0.90,2.44	0.119	Random-effects model
AA+AG vs. GG		0.626	0.00%	1.39	0.66,2.93	0.394	Fixed-effects model
AA vs.AG+GG		0.039	69.1%	1.50	0.90,2.52	0.122	Random-effects model
AA vs. AG		0.057	65.0%	1.46	0.89,2.39	0.134	Random-effects model

Stratification was performed by cancer type, and a significant higher risk correlation between rs2279115 and cancer susceptibility was found in digestive system cancers and in allelic, dominant, recessive and additive models(OR= 1.31, 95% CI:1.18,1.45, P= 0.000, allelic model respectively), but not in breast Cancer (OR= 1.05, 95%CI:0.87,1.28, P=0.599, allelic model respectively), respiratory cancer (OR= 1.32, 95%CI:0.89,1.97, P=0.170, allelic model respectively) and Hematopoietic cancer(OR=1.19, 95%CI:0.78,1.81, P=0.418, allelic model respectively). Furthermore, we found rs2279115 was correlated with a significantly higher risk in endocrine system cancer risk in allelic (OR=1.34, 95%CI:1.06,1.71, P=0.016) and recessive model(OR=1.49, 95%CI:1.06,2.09, P=0.023), but not in dominant (OR=1.45, 95%CI:0.57,3.71, P=0.439) and additive model(OR=1.42, 95%CI:0.99,2.04, P=0.058). This is may because lack of large sample size in endocrine system cancer. Therefore, consider the inconsistency of these results, large sample size is needed in rs2279115 polymorphism and cancer susceptibility in different cancer type. Table 2 displays the results of subgroup analysis in cancer type.

**Table 2:** Meta-analysis of rs2279115 polymorphism and cancer risk in cancer type

rs2279115	No.ofstudies	*P*_Q_	I^2^	OR	95% CI	*P*_Z_	Model
**Cancer type**							
**Hematopoietic cancer**	**2(333/333)**						
C vs. A		0.144	53.1%	1.19	0.78,1.81	0.418	Random-effects model
CC+ CA vs. AA		0.586	0.00%	0.99	0.63,1.57	0.978	Fixed-effects model
CC vs. CA+AA		0.130	56.4%	1.40	0.75,2.63	0.296	Random-effects model
CC vs. CA		0.136	54.9%	1.48	0.74,2.82	0.272	Random-effects model
**Digestive system cancers**	**3(1628/1640)**						
C vs. A		0.610	0.00%	1.31	1.18,1.45	<0.001	Fixed-effects model
CC+ CA vs. AA		0.371	0.00%	1.30	1.07,1.58	0.008	Fixed-effects model
CC vs. CA+AA		0.756	0.00%	1.51	1.31,1.74	<0.001	Fixed-effects model
CC vs. CA		0.701	0.00%	1.47	1.27,1.72	<0.001	Fixed-effects model
**Endocrine system cancer**	**2(210/435)**						
C vs. A		0.203	38.3%	1.34	1.06,1.71	0.016	Fixed-effects model
CC+ CA vs. AA		0.045	75.1%	1.45	0.57,3.71	0.439	Random-effects model
CC vs. CA+AA		0.649	0.00%	1.49	1.06,2.09	0.023	Fixed-effects model
CC vs. CA		0.856	0.00%	1.42	0.99,2.04	0.058	Fixed-effects model
**Breast Cancer**	**4(1470/1628)**						
C vs. A		0.082	55.3%	1.05	0.87,1.28	0.599	Random-effects model
CC+ CA vs. AA		0.005	76.5%	1.16	0.69,1.96	0.577	Random-effects model
CC vs. CA+AA		0.574	0.00%	1.07	0.92,1.25	0.374	Fixed-effects model
CC vs. CA		0.893	0.00%	1.03	0.87,1.21	0.742	Fixed-effects model
**Respiratory cancer**	**3(1537/2057)**						
C vs. A		0.000	92.6%	1.32	0.89,1.97	0.170	Random-effects model
CC+ CA vs. AA		0.001	84.7%	1.46	0.82,2.58	0.198	Random-effects model
CC vs. CA+AA		0.000	91.6%	1.45	0.86,2.46	0.163	Random-effects model
CC vs. CA		0.000	86.9%	1.37	0.88,2.14	0.163	Random-effects model

Stratification was performed by ethnicity, rs2279115 was correlated with a significantly higher risk of cancer susceptibility in Asia (OR= 1.28, 95% CI:1.11,1.48, P= 0.001, allelic models respectively) but not in Caucasian (OR=1.01, 95% CI:0.85,1.21, P=0.879, allelic models respectively) in allelic, dominant, recessive, and additive models. Simultaneously, rs1801018 had no correlation with the risk of cancer susceptibility in Asia in allelic, dominant, recessive, and additive models(OR= 1.48, 95% CI:0.90,2.44, P= 0.119, allelic model respectively). There is lack of data in Caucasian. Table 3 displays the results of subgroup analysis in ethnicity.

**Table 3:** Meta-analysis of rs2279115 polymorphism and cancer risk in ethnicity

rs2279115	No.ofstudies	*P*_Q_	I^2^	OR	95% CI	*P*_Z_	Model
**Ethnicity**							
**Asia**	**11(3869/4717)**						
C vs. A		0.000	76.2%	1.28	1.11,1.48	0.001	Random-effects model
CC+ CA vs. AA		0.000	70.6%	1.39	1.07,1.82	0.014	Random-effects model
CC vs. CA+AA		0.000	70.9%	1.39	1.16,1.67	<0.001	Random-effects mode
CC vs. CA		0.005	60.0%	1.33	1.13,1.57	0.001	Random-effects mode
**Caucasian**	**7(3081/3267)**						
C vs. A		0.075	56.6%	1.01	0.85,1.21	0.879	Random-effects mode
CC+ CA vs. AA		0.076	56.4%	1.01	0.74,1.40	0.931	Random-effects mode
CC vs. CA+AA		0.337	11.2%	1.03	0.89,1.19	0.710	Fixed-effects model
CC vs. CA		0.709	0.00%	1.02	0.87,1.19	0.814	Fixed-effects model

### Meta-analysis of BCL-2 promoter polymorphism and cancer prognosis

Overall, our results showed that rs2279115 polymorphism was significantly correlated with the cancer prognosis in all four genetic models (HR=1.09, 95% CI:1.03,1.51, P= 0.000, allelic models respectively). Stratification was performed by ethnicity, rs2279115 was correlated with a significantly higher risk of cancer prognosis in Asia (HR= 1.17, 95% CI:1.01,1.41, P= 0.000, allelic models respectively) but not in Caucasian (HR=1.01, 95%CI:0.90,1.41, P=0.878, allelic models respectively). Simultaneously, there is no correlation between rs1801018 and cancer prognosis (HR=95, 95%CI:0.68,1.36, P=0.547). Table 4 displays the results of analysis.

**Table 4:** Meta-analysis of BCL-2 promoter polymorphisms and cancer prognosis

Genetic model	No.ofstudies	*P*_Q_	I^2^	HR	95% CI	*P*_Z_	Model
**rs2279115**							
**Total**	**13(4013/5319)**						
CC vs. CA		0.011	59.6%	1.09	1.03,1.51	<0.001	Random-effects model
CC vs. AA		0.018	58.5%	1,18	1.07,1.66	<0.001	Random-effects model
CA vs. AA		0.677	0.00%	1.31	1.12,2.61	<0.001	Fixed-effects model
CC vs. CA+AA		0.000	84.8%	1.26	1.16,1.71	<0.001	Random-effects model
**Asia**	**6(2813/3124)**						
CC vs. CA		0.000	55.5%	1.17	1.01,1.41	<0.001	Random-effects model
CC vs. AA		0.000	38.4%	1.50	1.01,2.15	<0.001	Fixed-effects model
CA vs. AA		0.000	55.1%	1.38	1.03,1.87	<0.001	Random-effects model
CC vs. CA+AA		0.000	53.4%	1.21	1.06,1.75	<0.001	Random-effects model
**Caucasian**	**7(1200/2195)**						
CC vs. CA		0.651	0.0%	1.01	0.90,1.13	0.878	Fixed-effects model
CC vs. AA		0.392	5.2%	1.04	0.89,1.21	0.937	Fixed-effects model
CA vs. AA		0.299	15.2%	1.07	0.92,1.45	0.430	Fixed-effects model
CC vs. CA+AA		0.180	29.9%	1.35	0.72,2.52	0.829	Fixed-effects model
**rs1801018**	**5(1889/2110)**						
AA vs. AG+GG		0.088	43.6%	0.95	0.68,1.36	0.547	Fixed-effects model
AG vs. GG		0.610	0.0%	1.51	0.74,2.13	0.429	Fixed-effects model

### Sensitivity analysis

Sensitivity analysis was conducted to assess the stability of the results. The results show rs2279115 in four genetic model were stable in Supplementary Figure 1-4, and rs1801018 in four genetic model were stable in Supplementary Figure 5-8.

### Publication bias

Each studies in this meta-analysis were performed to evaluate the publication bias by both Begg's funnel plot and Egger's test. P>0.05 was considered no publication bias. The results show no obvious evidence of publication bias was found in allelic, dominant, recessive or additive genetic model in rs2279115 and rs1801018 in Table 5.

**Table 5:** Publication bias analysis of the meta-analysis

Genetic model	Test	t	95% CI	P
**rs2279115**				
C vs. A	Begg's test			0.120
	Egger's test	-2.73	-9.59,-1.21	0.107
CC+ CA vs. AA	Begg's test			0.272
	Egger's test	-4.09	-0.84,-027	0.125
CC vs. CA+AA	Begg's test			0.472
	Egger's test	3.27	1.11,5.21	0.231
CC vs. CA	Begg's test			0.791
	Egger's test	1.85	-0.48,6.99	0.403
**rs1801018**				
A vs. G	Begg's test			0.602
	Egger's test	-4.82	-2.60,1.17	0.130
AA+AG vs. GG	Begg's test			0.117
	Egger's test	-9.04	-0.09,0.02	0.070
AA vs.AG+GG	Begg's test			0.602
	Egger's test	-5.03	-3.15,1.36	0.125
AA vs. AG	Begg's test			0.602
	Egger's test	-5.22	-2.82,1.17	0.121

## DISCUSSION

Bcl-2 is an important anti-apoptotic protein that can regulate cell death and is thus classified as an oncogene [48]. There is increasing evidence that Bcl-2 gene polymorphism may be associated with cancer susceptibility and prognosis. Recently,polymorphism in Bcl-2 gene, variant in promoter region rs2279115 and rs1801018, have been reported to be associated with cancer susceptibility and prognosis many times. Whether Bcl-2 promoter polymorphisms are related to cancer susceptibility and prognosis, however, the results are incompatible. This might be the first meta-analysis regarding Bcl-2 promoter polymorphisms in cancer susceptibility and prognosis.

In this study, we found that rs2279115 have a significantly higher risk of cancer susceptibility and prognosis in allelic, dominant, recessive, and additive models. However, rs1801018 had no associated with cancer susceptibility and prognosis in allelic, dominant, recessive, and additive model. Subgroups analysis indicated that rs2279115 was associated with a significantly higher risk of cancer in Asia but not in Caucasian. Furthermore, rs2279115 was associated with a significantly higher risk of cancer in digestive system cancer and endocrine system cancer but not in breast cancer, respiratory cancer and hematopoietic cancer. This is may because lack of large sample size in breast cancer, respiratory cancer and hematopoietic cancer. Therefore, consider the inconsistency of these results, large sample size is needed in rs2279115 polymorphism and cancer susceptibility in different cancer type. Simultaneously,rs2279115 was correlated with a significantly higher risk of cancer prognosis in Asia but not in Caucasian. Considering these promising results, rs2279115 may be a tumor marker for cancer therapy in Asia.

Although, we performed this meta-analysis very carefully, however, some limitations must be considered in the current meta-analysis. First, we performed stratification only by ethnicity and cancer type, without referring other factors. Further research should be conducted in other cancer type and other ethnicity population. Second, we only select literature that written by English, other language should be chosen in the further. Third, in the subgroup analysis in cancer type, there might be insufficient statistical power to check an association.

In conclusion, our meta-analysis suggests a role BCL-2 promoter polymorphisms in cancer susceptibility and prognosis, rs2279115 but not rs1801018 may be a tumor marker for cancer therapy in Asia. However, large sample size, different ethnic population and different cancer type is warranted to validate this association.

## MATERIALS AND METHODS

### Literature search

We searched PubMed and Embase databases up to August 30, 2016, with keywords including “cancer” and “BCL-2 or B cell lymphoma-2” and “single nucleotide polymorphism or mutation or variation or SNP”. We also manually checked reference lists to identify other potential studies and restricted the search to human studies. The database search was performed independently by Binhui Yang and Zhongqiu Liu and the disagreements were resolved through consensus by all of the authors.

### Inclusion and exclusion criteria

If the following inclusion were satisfied, studies would be included in our meta-analysis: 1) case-control studies focused on association between the Bcl-2 promoter polymorphism and cancer susceptibility or prognostic significance. 2) Studies provided sufficient data to estimate the odds ratio (OR) or hazard ratio (HR) and 95% confidence intervals (CI) according to Bcl-2 promoter polymorphisms. 3) When study patients overlapped with patients in other included studies, we selected the first study published. The two researchers (Wei Li and Qihua He) read the titles and abstracts independently and excluded the uncorrelated studies; then the full-texts were examined by our review team and the disagreements were resolved through consensus by all of the authors. The studies would be selected according to the inclusion criteria.

### Data extraction

The following information in studies investigating the association between Bcl-2 promoter polymorphisms and susceptibility was extracted by two independent researchers: (1) first author; (2) publication year; (3) mean value of age; (4) country and ethnicity; (5) cancer type; (6) cases and controls sample size; (7)genotype. As for studies investigating the association between Bcl-2 polymorphism and cancer prognostic, two researchers independently extracted the following information from the article:(1) first author; (2) publication year; (3) mean value of age; (4) country and ethnicity; (5) cancer type; (6) cases and controls sample size; (7) genotype; (8) HR estimation. The two researchers (Wei Li and Qihua He) read the reports independently, and the disagreements were resolved through consensus by all of the authors.

### Statistical analysis

STATA software 12.0 (STATA Corp, College Station, TX, USA) were used to evaluate the relationships between Bcl-2 promoter polymorphisms and cancer susceptibility and prognosis. Studies were assessed by chi-square in control group under Hardy-Weinberg equilibrium (HWE) to calculate frequencies of BCL-2, and if P< 0.05, study was considered to be disequilibrium. The strength of the relationship between Bcl-2 polymorphisms including rs2279115 and rs1801018 and cancer susceptibility were evaluated by odd ratios (ORs) with corresponding 95% confidence intervals (CIs). The correlation between Bcl-2 polymorphisms and cancer prognosis were measured by hazard ratios (HRs). By using Q test and I^2^ statistic to assess heterogeneity among studies in rs2279115 in the allelic (C vs. A), dominant (CC+ CA vs. AA), recessive (CC vs. CA+AA), and additive (CC vs. CA) and in rs1801018 in the allelic (A vs. G), dominant (AA+AG vs. GG), recessive (AA vs.AG+GG), and additive (AA vs. AG) genetic models. Random-effect model was chosen if P_Q_< 0.10 or I^2^>50%, otherwise, fixed-effect mode was applied. Sensitivity analysis was conducted to assess the stability of the results. Begg's and Egger's tests were to assess the publication bias of each study. P< 0.05 was considered signifcant for all tests.

## SUPPLEMENTARY MATERIALS FIGURES AND TABLES




