# Supplementary material for: Genetic polymorphisms of Bcl-2 promoter in cancer susceptibility and prognosis: a meta-analysis
Source: Oncotarget. 2017 Feb 27;8(15):25270–8. doi: 10.18632/oncotarget.15751 (PMC5421928; doi:10.18632/oncotarget.15751)
Supplement: Supplementary file 1 [file oncotarget-08-25270-s001.pdf]

Genetic polymorphisms of Bcl-2 promoter in cancer susceptibility and prognosis: a meta-analysis

SUPPLEMENTARY MATERIALS

SUPPLEMENTARY FIGURES AND TABLES

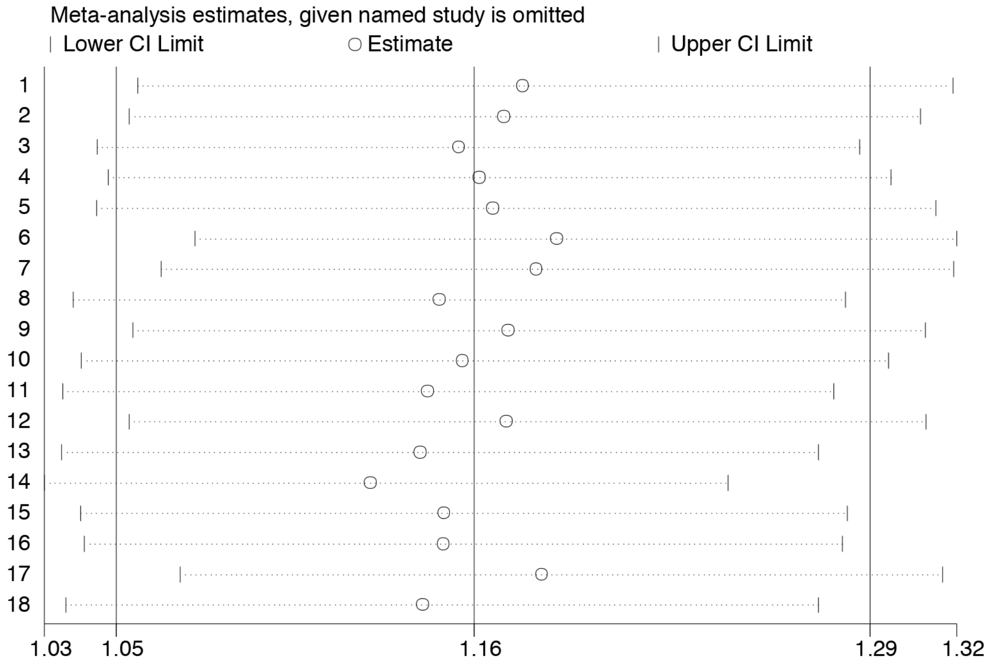

Supplementary Figure 1: Forest plots of sensitivity analysis for the meta-analysisn of rs2279115 and cancer risk in allelic (C vs. A) model.

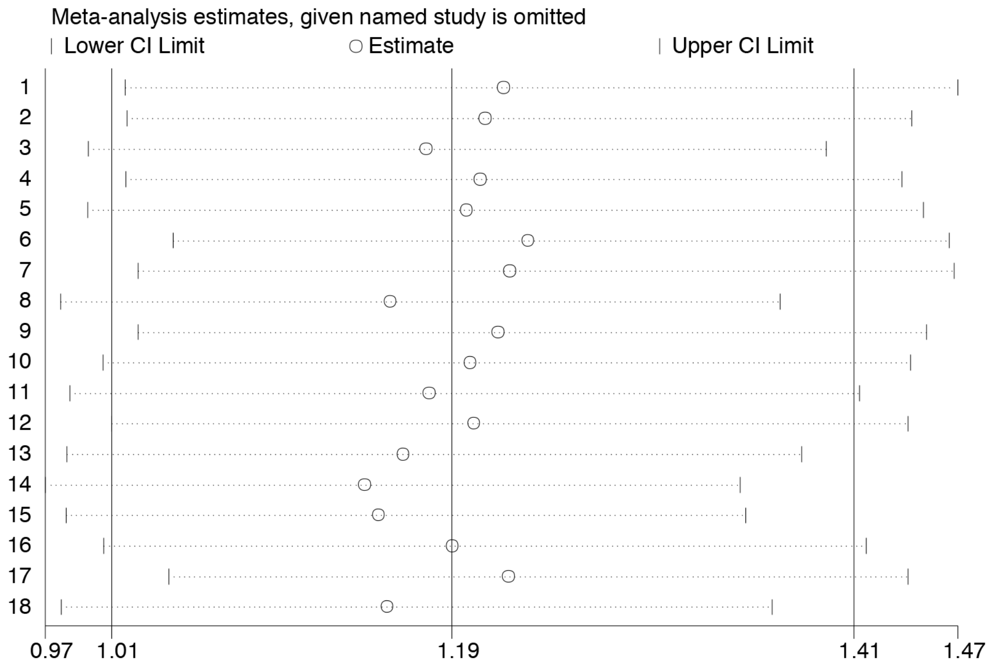

Supplementary Figure 2: Forest plots of sensitivity analysis for the meta-analysisn of rs2279115 and cancer risk in dominant (CC+ CA vs. AA) model.

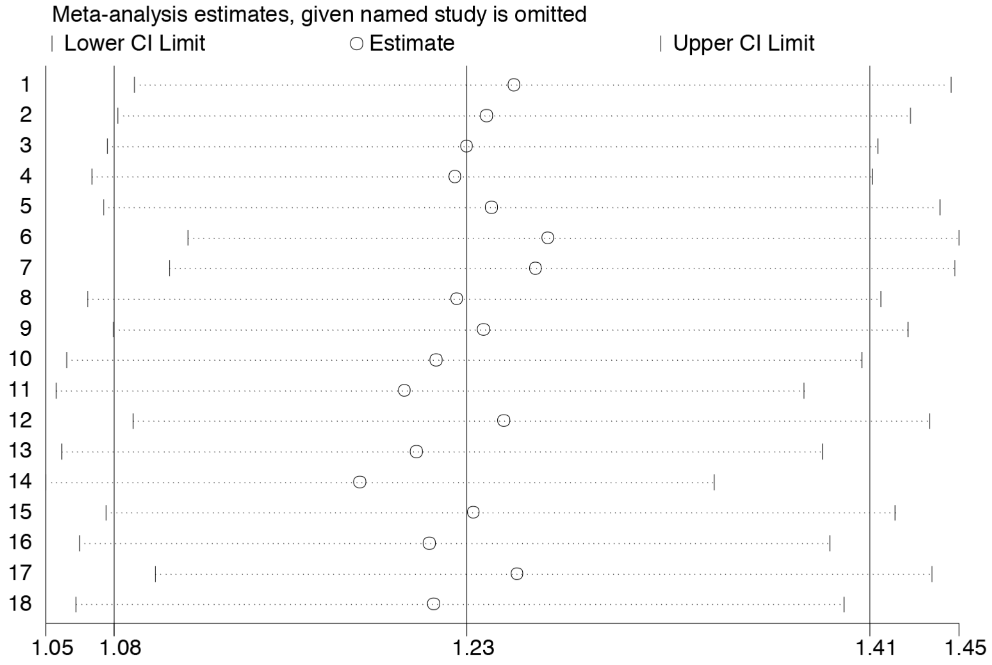

Supplementary Figure 3: Forest plots of sensitivity analysis for the meta-analysisn of rs2279115 and cancer risk in recessive (CC vs. CA+AA) model.

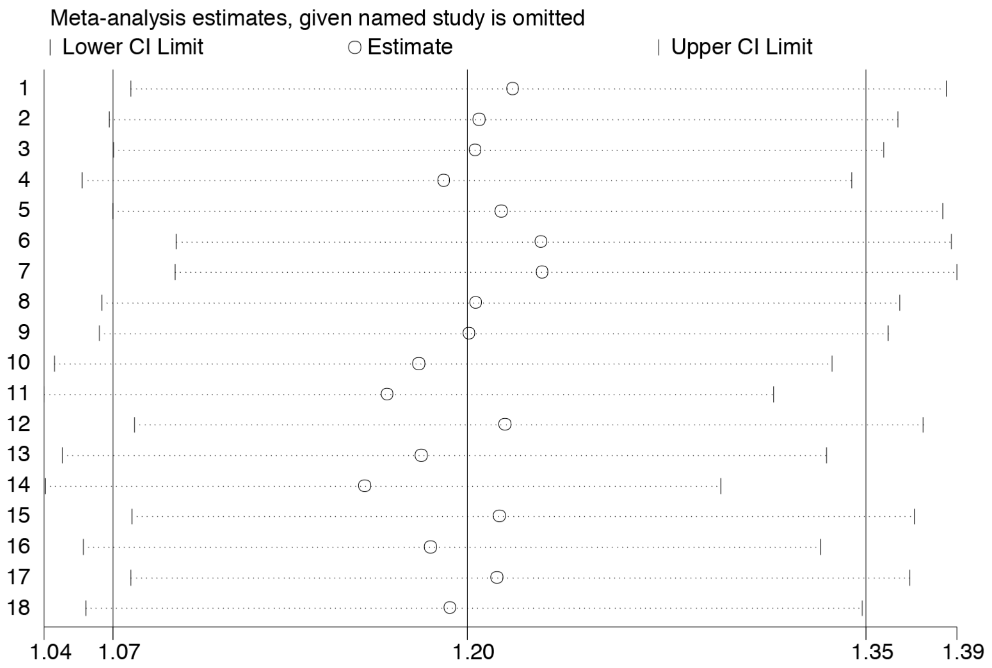

Supplementary Figure 4: Forest plots of sensitivity analysis for the meta-analysisn of rs2279115 and cancer risk in additive (CC vs. CA) model.

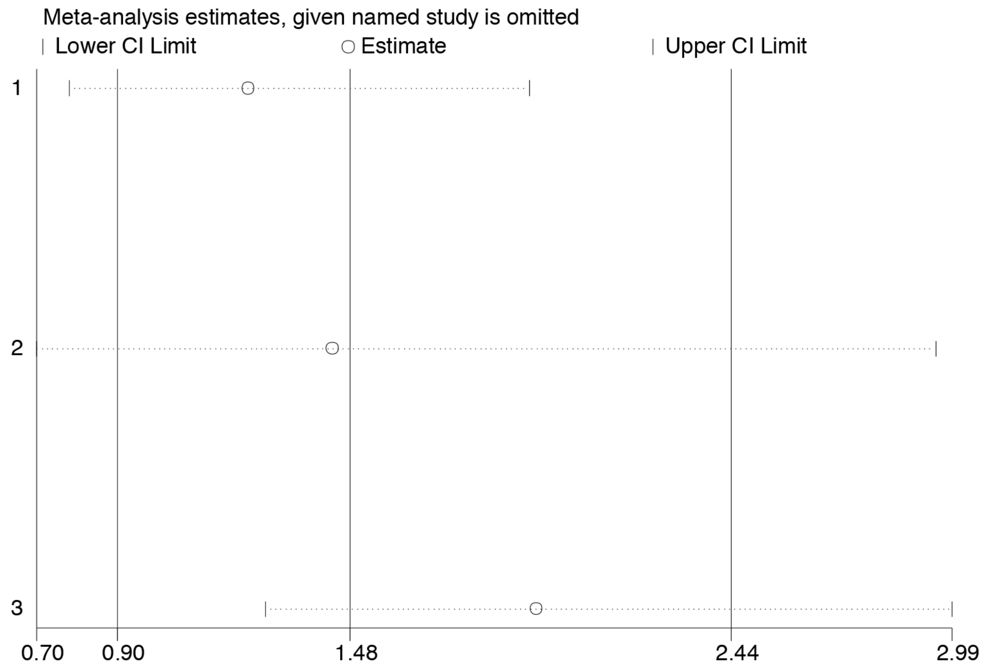

Supplementary Figure 5: Forest plots of sensitivity analysis for the meta-analysisn of rs1801018 and cancer risk in allelic (A vs. G) model.

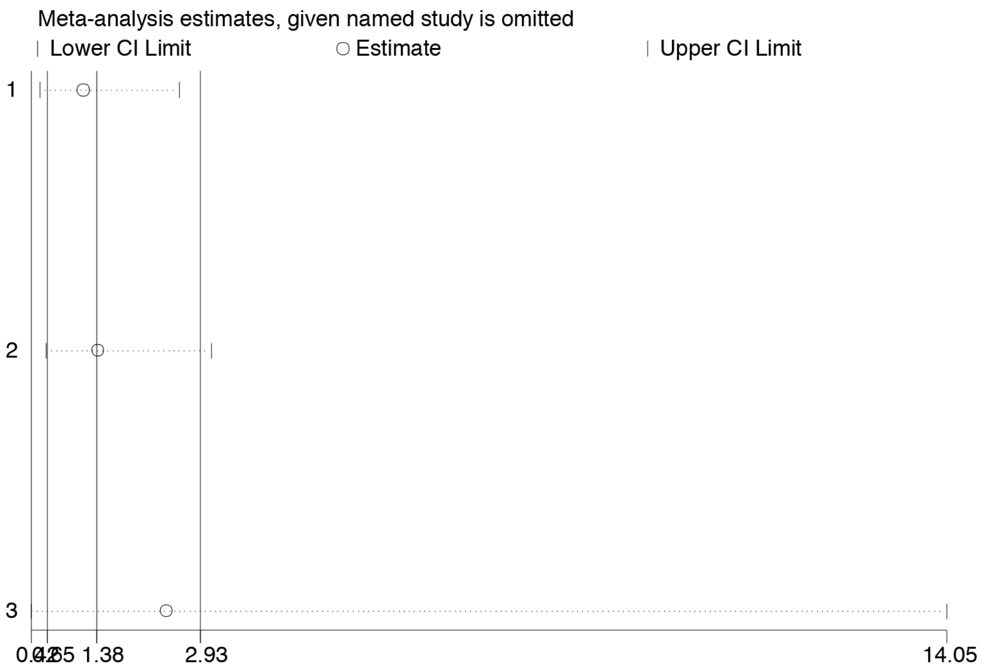

Supplementary Figure 6: Forest plots of sensitivity analysis for the meta-analysisn of rs1801018 and cancer risk in dominant (AA+AG vs. GG) model.

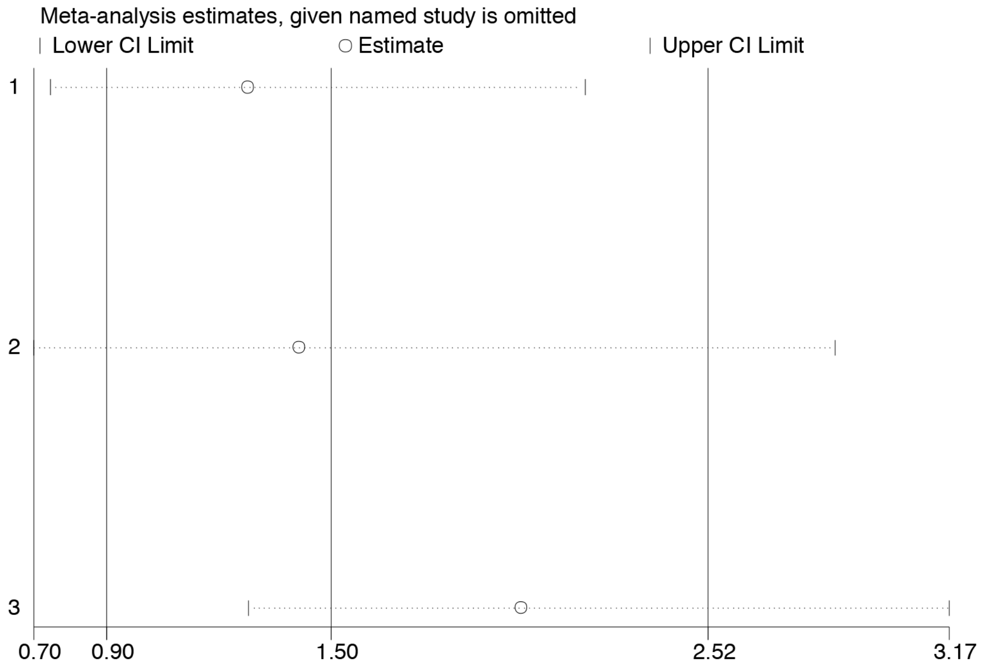

Supplementary Figure 7: Forest plots of sensitivity analysis for the meta-analysisn of rs1801018 and cancer risk in recessive (AA vs.AG+GG) model.

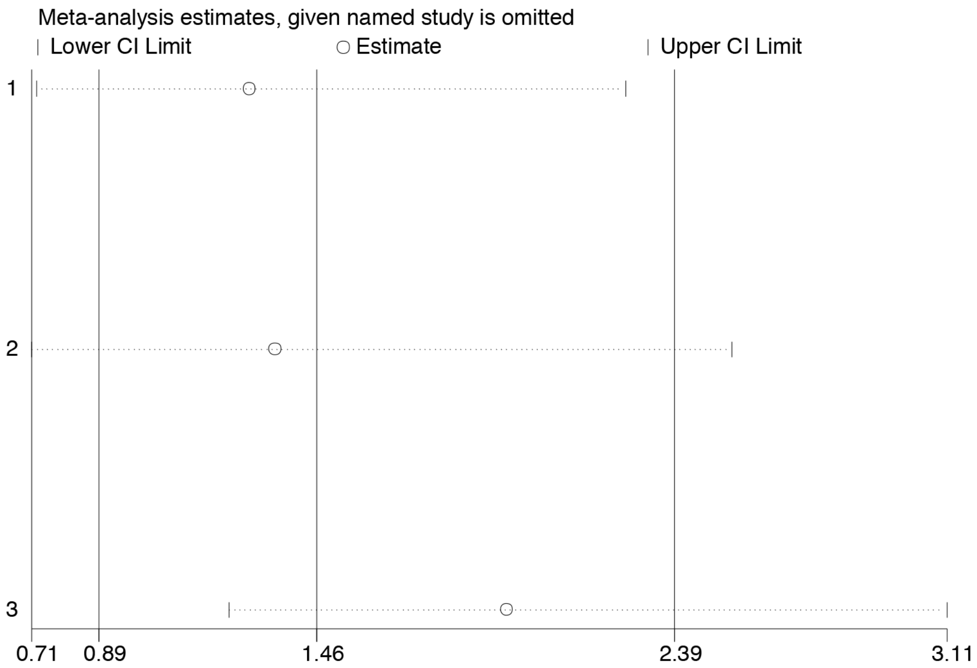

Supplementary Figure 8: Forest plots of sensitivity analysis for the meta-analysisn of rs1801018 and cancer risk in additive (AA vs. AG) model.

Supplementary Table 1: Characteristics of studies included in this meta-analysis.

See Supplementary File 1

Supplementary Table S2: Genotype frequencies of rs2279115 included in this meta-analysis

Author	Case			Control			MAF		HWE
	CC	CA	AA	CC	CA	AA	Case	Control	
Chen K	226	382	206	257	446	231	0.51	0.51	0.18
Zenz T	123	194	73	36	63	21	0.45	0.56	0.47
Fingas CD	14	21	5	12	18	10	0.61	0.53	0.54
Eun YG	40	37	15	80	109	33	0.64	0.61	0.68
Liu Z	75	96	34	96	112	16	0.60	0.68	0.03
Searle CJ	314	477	189	290	475	216	0.56	0.54	0.41
Meyer A	146	259	104	152	238	76	0.54	0.58	0.29
Xu P	378	483	156	393	479	145	0.61	0.62	0.96
Wang WL	147	198	79	191	223	32	0.58	0.68	0.002
Li W	128	105	19	109	103	36	0.72	0.65	0.15
Oliveira C	64	90	46	64	107	44	0.55	0.55	0.95
Cingeetham A	144	47	30	229	37	39	0.76	0.81	0.00
Pan W(H)	242	265	80	198	312	90	0.64	0.59	0.07
Pan W(J)	416	453	131	312	516	172	0.64	0.57	0.10
Fernandes AT	67	111	53	83	132	68	0.53	0.53	0.28
Yang X(H)	92	92	16	136	208	56	0.69	0.60	0.10
Yang X(J)	136	146	38	170	336	134	0.65	0.53	0.18
Bhushann Meka P	116	81	7	58	38	14	0.77	0.70	0.06
Moazami-Goudarzi M	33	18	11	22	27	13	0.68	0.57	0.39
Mou X	90	95	15	52	50	27	0.69	0.60	0.03
Zhang Ning	42	53	19	46	53	8	0.60	0.68	0.17
Wang Ying-Xue	92	102	19	38	58	22	0.67	0.57	0.21
Hirata H	92	83	41	101	72	36	0.62	0.66	0.00
Hirata H	56	67	17	80	62	25	0.64	0.66	0.03

Supplementary Table 3: Genotype frequencies of rs1801018 included in this meta-analysis

Author	Case			Control			MAF		HWE
	AA	AG	GG	AA	AG	GG	Case	Control	
Meenu Jain	135	16	0	160	38	3	0.94	0.89	0.67
Eun YG	79	12	1	170	49	3	0.92	0.88	0.80
Liu Z	189	11	5	194	24	6	0.95	0.92	0.00
Xu P	855	152	10	864	159	12	0.92	0.91	0.13
Wang WL	376	35	13	377	48	21	0.93	0.90	0.00
