# Supplementary material for: Genetic polymorphisms of Bcl-2 promoter in cancer susceptibility and prognosis: a meta-analysis
Source: Oncotarget. 2017 Feb 27;8(15):25270–8. doi: 10.18632/oncotarget.15751 (PMC5421928; doi:10.18632/oncotarget.15751)
Supplement: Supplementary file 2 [file oncotarget-08-25270-s002.docx]

**Table S1:Characteristics of studies included in this meta-analysis**

|  | **Year** | **Mean Age**  **Case/Control** | **Cancer type** | **Country** | **Ethnicity** | **Sample size** | **Polymorphism** |
| --- | --- | --- | --- | --- | --- | --- | --- |
|  |  |  |  |  |  | **Case/****Control** |  |
| Chen K | 2007 | 57.0/55.9 | squamous cell carcinoma of the head and neck | USA | America | 814/934 | rs2279115 |
| Fingas CD | 2010 | 62.1/61.3 | Extrahepatic Cholangiocarcinoma | German | Europe | 40/40 | rs2279115 |
| Eun YG | 2011 | 53.5/53.8 | Papillary Thyroid Cancer | Korean | Asia | 92/222 | rs2279115  rs1801018 |
| Liu Z | 2012 | 62.3/63.4 | esophageal cancer | China | Asia | 205/224 | rs2279115  rs1801018 |
| Searle CJ | 2012 | 59.0/57.0 | breast cancer | UK | Europe | 1015/1034 | rs2279115 |
| Meyer A | 2013 | 66.3/39 | prostate cancer | German | Europe | 510/490 | rs2279115 |
| Xu P | 2013 | 60.3/59.7 | Lung Cancer | China | Asia | 1017/1017 | rs2279115  rs1801018 |
| Wang WL | 2014 | 54.2/55.3 | developing non-Hodgkin lymphoma | China | Asia | 424/446 | rs2279115  rs1801018 |
| Li W | 2014 | 52.3/54.2 | glioma | China | Asia | 248/252 | rs2279115 |
| Oliveira C | 2014 | 55.0/52.0 | cutaneous melanoma | Brazil | America | 200/215 | rs2279115 |
| Cingeetham A | 2015 | 30.76/31.4 | Acute myeloid leukemia | India | Asia | 221/305 | rs 2279115 |
| Pan W(H) | 2015 | 56.3/53.2 | esophageal squamous cell carcinoma | China | Asia | 588/600 | rs 2279115 |
| Pan W(J) | 2015 | 57.4/56.1 | esophageal squamous cell carcinoma | China | Asia | 1000/1000 | rs2279115 |
| Fernandes AT | 2015 | 37.8/46.2 | cervical cancer | Brazil | America | 231/283 | rs2279115 |
| Yang X(H) | 2016 | 57.1/58.3 | small cell lung cancer | China | Asia | 200/400 | rs2279115 |
| Yang X(J) | 2016 | 56.2/57.3 | small cell lung cancer | China | Asia | 320/640 | rs2279115 |
| Bhushann Meka P | 2016 | 41.2/40.1 | breast cancer | India | Asia | 110/204 | rs2279115 |
| Moazami-Goudarzi M | 2016 | 7.4/7.2 | acute lymphoblastic leukemia | Iran | Asia | 62/62 | rs2279115 |
| Mou X | 2015 | NA | Gastric cancer | China | Asia | 200/134 | rs2279115 |
| Zhang Ning | 2012 | 49.1/47.8 | breast cancer | China | Asia | 114/107 | rs2279115 |
| Wang Ying-Xue | 2011 | 51.3/54.2 | papillary thyroid carcinoma | China | Asia | 118/213 | rs2279115 |
| Zenz T | 2009 | 56/52 | chronic lymphocytic leukemia | German | Asia | 271/271 | rs2279115 |
| Hirata H | 2009 | 62.2/61.6 | Renal Cancer | Japan | Asia | 216/209 | rs2279115 |
| Hirata H | 2009 | 68.0/68.0 | Prostatectomy Cancer | Japan | Asia | 140/167 | rs2279115 |
